# Supplementary material for: VEGF blockade enhances the antitumor effect of BRAFV 600E inhibition
Source: EMBO Mol Med. 2016 Dec 14;9(2):219–37. doi: 10.15252/emmm.201505774 (PMC5286370; doi:10.15252/emmm.201505774)
Supplement: Supplementary file 1 — Appendix [file EMMM-9-219-s001.pdf]

Appendix Comunanza V et al. (EMM-2015-05774)

- Appendix Supplementary Legends
- Appendix Supplementary Tables S1 to S6
- Appendix Supplementary Figure S1 to S6

Appendix Supplementary Figure Legends

Appendix Figure S1. Effect of the combination of PLX4720 and bevacizumab on apoptosis of A375 cells transplanted in athymic nude mice.

Representative images of tumor cell apoptosis determined by immunofluorescence staining with M30-cytodeath antibody in A375 xenografts treated as indicated. Bar graphs indicate the $M30^{+}$ area/tumor area (n= 3 tumors), ***P = < 0.001 versus vehicle (P = 9.71E-05).
The scale bars represent 50 μm. The results are given as the mean ± SEM. Significance was assessed by one-way ANOVA test followed by post-hoc pairwise analysis test.

Appendix Figure S2. Vascular response of COLO205 and A375 xenograft tumors to PLX4720 and bevacizumab.
(A) Representative images of vasculature determined by CD31 immunofluorescence staining in COLO205 xenografts treated as indicated. Bar graphs indicate quantitative micro vessel density (MVD) and micro vessel area (MVA) analysis (n=6 tumors), *P < 0.05, **P < 0.01, ***P < 0.001 versus vehicle (MVD: PLX4720 P = 0.0002; bevacizumab P = 0.025; COMBO P = 4.48E-09) (MVA: PLX4720 P = 0.0080 ; bevacizumab P = 0.046 ; COMBO P = 0.025).
(B) Histogram showing the vessel size distribution in A375 xenografts treated as indicated (n=6 tumors) *P < 0.05, **P < 0.01, ***P < 0.001 versus vehicle (<100 μm^2: PLX4720 P = 0.001, COMBO P = 0.017), (500-600 μm^2 : PLX4720 P = 0.022, COMBO P = 0.028), (>600 μm^2 : PLX4720 P = 0.00058, COMBO P = 0.0032).
(C) Representative images of pericyte coverage determined by CD31 (green) and NG2 (red) immunofluorescence co-staining in A375 xenografts treated as indicated. Bar graphs indicate the $CD31^{+}NG2^{+}$area/tumor area in A375 xenografts (n=5 tumors).
The scale bars represent 100 μm in (A) and 50 μm in (C). The results are given as the mean ± SEM. Significance was assessed by one-way ANOVA test followed by post-hoc pairwise analysis test (A-B-C).

Appendix Figure S3. Transcriptional profiling of Human genes in A375 xenografts in response to PLX4720, bevacizumab and COMBO.

(A) Heatmap showing unsupervised hierarchical clustering of human differentially expressed modulated genes in the vehicle, PLX4720, bevacizumab and COMBO treatments in A375 melanoma xenograft tumors.

(B) Volcano plots of human gene expression showing fold-change and p-value for the comparison of PLX4720 (*left*), bevacizumab (*middle*) and COMBO (*right*) treatments versus vehicle. Up-regulated and down-regulated genes are colored red and blue, respectively.

(C) Summary of the functional categories of human genes significantly enriched in response to PLX4720 and COMBO. GO analyses were performed individually on down- or up-regulated genes using DAVID tools (biological process). GO terms are ranked by p-value corrected by BH method, and the number of genes is indicated. For a complete list of significantly enriched GO groups, see Tables SI-SII.

Appendix Figure S4. Transcriptional profiling of murine genes in A375 xenografts in response to PLX4720, bevacizumab and COMBO.

(A) Heatmap showing unsupervised hierarchical clustering of murine differentially modulated genes in the vehicle, PLX4720, bevacizumab and COMBO treatments in A375 melanoma xenograft tumors.

(B) Modulation of genes related to angiogenesis after bevacizumab treatment by microarray analysis.

(C) Quantitative PCR analysis of the expression of angiogenesis-related genes in bevacizumab-treated tumors in A375 xenografts. The data are presented as the mean ± SEM of expression fold-change (log_2) compared with vehicle after normalization to the housekeeping gene (n=3), $^*P < 0.05$; $^{**}P < 0.01$; $^{***}P < 0.001$ versus vehicle (Pecam P = 0.026; Flt-1 P = 0.036; Cdh55 P = 0.0021; Pdgfb P = 0.0029; Col4a1 P = 0.018; Col4a2 P = 0.0030; Hey P = 5.31E-05; Ptn P = 0.00019).

(D) Volcano plots of murine gene expression showing fold-change and p-value for the comparison of PLX4720 (*left*), bevacizumab (*middle*) and COMBO (*right*) treatments versus vehicle. Up-regulated and down-regulated genes are colored red and blue, respectively.

(E) Heatmap representation of gene expression changes within the “Reactome Cytokine” “Signaling in Immune System”, “Innate Immune System” gene set. Genes in heatmaps are shown in rows and samples in columns. Expression level is represented as a gradient from

high (red) to low (blue). V, P, B and C respectively indicate vehicle, PLX4720, bevacizumab and COMBO.

(F) IPA interaction networks of “Immune Response” and “ECM remodeling” modulated genes comparing COMBO- and vehicle-treated tumors. The solid lines correspond with all direct interactions in the IPA database. The dashed lines represent indirect interactions. Genes that were either up-regulated (red nodes) or down-regulated (blue nodes) are integrated in the signaling network.

Appendix Figure S5. **Analysis of myeloid subpopulations in A375 xenografts.**

(A) Analysis of h-GMCSF and TNFA gene expression. Real time quantitative PCR of tumors treated with PLX4720, bevacizumab or COMBO. The data are presented as the mean ± SEM of the fold change in expression (log_2) compared with vehicle after normalization for the housekeeping gene TBP (n=3), *P < 0.05; **P < 0.01 (h-GMCSF: PLX4720 P = 0.0038, COMBO P = 0.0040; hTNFA: PLX4720 P = 0.016, COMBO P = 0.0018).

(B) Representative images of NEMs in a vehicle-treated A375 xenograft determined by CD11b, Nrp1 and Gr1 immunofluorescence staining. Bar graph indicate $CD11b^+ Gr1^+ Nrp1^+$ area/tumor area in xenografts treated as indicated (n = 5 tumors), ***P < 0.001 versus vehicle (P = 1.56E-05)

The scale bars represent 50 μm in (B). The results are given as the mean ± SEM. Significance was assessed by and Student’s *t*-test (A) and one-way ANOVA test followed by post-hoc pairwise analysis test (B).

Appendix Figure S6. Effect of long-term treatment of A375 xenografts with PLX4720, bevacizumab or COMBO.

(A) Mice bearing established A375 were treated with vehicle (n=5), PLX4720 (n=6) bevacizumab (n=5) or COMBO (n=6) for 6 weeks. Tumor growth is expressed as % change of the initial tumor, *P < 0.05, **P < 0.01 ***P < 0.001 versus vehicle (PLX4720: 2 week P = 0.0009; 3 week P = 0.014), (COMBO: 2 week P = 6.27E-05; 3 week P = 0.0006; 4 week P = 0.0020; 5 week P = 0.030).

(B) Representative images of a responder COMBO-treated tumor sections co-stained with an antibody against CD68 and an antibody anti-CCR7 (M1-like polarization) or anti-C206 (M2-like polarization).

(C) Analysis of the expression of h-GMCSF in COMBO-responder and -relapsing mice. The data are presented as the mean ± SEM of expression fold change (log_2) compared with vehicle after normalization to the housekeeping gene TBP (n=3), *$P < 0.05$ ($P = 0.017$).

(D) Representative images of vasculature determined by CD31 immunofluorescence staining in a responder and a relapsing A375 xenografts. Bar graphs indicate quantitative micro vessel area (MVA) analysis (n=3 tumors).

(E) Representative images of hypoxia marker pimonidazole in a responder and a relapsing A375 xenografts. Bar graphs indicate the % of tumor hypoxic area (n=3 tumors), **$P < 0.01$ versus COMBO responders ($P = 0.0062$).

The scale bar represents 50 μm in (B) and 100 μm in (D-E). The results are presented as the mean value ± SEM. Significance was assessed by one-way ANOVA test followed by post-hoc pairwise analysis test (A) and Student's t-test (C-D-E).

Appendix Table S1. Gene Ontology analysis for Human genes highly modulated by PLX4720.

Up-Modulated

Category	GO Term	Genes	p value BH corrected
**GOTERM_BP_FAT**	GO:0006955 immune response	CXCL1, IFIH1, CCL2, ENPP2, IFITM2, IFITM3, OAS3, RSAD2, IFI44L, OAS1, OAS2, HLA-DMA, IFI35, CXCL10, CFH, SEMA3C, CFI, DHX58, BST2, IL8, PRG4, HLA-B, TRIM22, PSMB9, DDX58, OASL, IL18BP, CXCL16, IFI6	5.56E-08
**GOTERM_BP_FAT**	GO:0009615 response to virus	DDX58, IFIH1, BST2, ISG15, IRF7, RSAD2, MX1, TRIM22, IFI35, STAT2	5.28E-04
**GOTERM_BP_FAT**	GO:0009611 response to wounding	PLAT, CXCL1, A2M, CCL2, IL8, AIF1, TNC, COL3A1, NINJ2, TGFB3, BDKRB1, EPHA3, CXCL10, NUPR1, IRF7, CFH, VCAN, ID3, CFI	0.001030
**GOTERM_BP_FAT**	GO:0006952 defense response	CXCL1, A2M, IFIH1, CCL2, IL8, AIF1, RSAD2, BDKRB1, HLA-B, EPHA3, CXCL10, DDX58, NUPR1, CXCL16, IRF7, CFH, CFI, MX1, DHX58, GNG7	0.00158
**GOTERM_BP_FAT**	GO:0030198 extracellular matrix organization	LUM, COL3A1, COL1A2, OLFML2A, POSTN, DCN, COL1A1, EMILIN1	0.0134
**GOTERM_BP_FAT**	GO:0043062 extracellular structure organization	TNC, LUM, COL3A1, COL1A2, OLFML2A, POSTN, DCN, COL1A1, EMILIN1	0.03061
**GOTERM_BP_FAT**	GO:0022610 biological adhesion	SVEP1, CCL2, TNC, COL22A1, COL3A1, NINJ2, MGP, POSTN, PCDH7, EMILIN1, VCAM1, LAMA4, COL6A3, VCAN, COL8A1, THBS2, BOC, CDH10	0.04327
**GOTERM_BP_FAT**	GO:0006954 inflammatory response	CXCL1, A2M, CCL2, NUPR1, IL8, AIF1, IRF7, CFH, BDKRB1, CFI, CXCL10, EPHA3	0.04359
**GOTERM_BP_FAT**	GO:0007155 cell adhesion	SVEP1, CCL2, TNC, COL22A1, COL3A1, NINJ2, MGP, POSTN, PCDH7, EMILIN1, VCAM1, LAMA4, COL6A3, VCAN, COL8A1, THBS2, BOC, CDH10	0.04777

Down-Modulated

Category	GO Term	Genes	p value BH corrected

**GOTERM_BP_FAT**	GO:0019318 hexose metabolic process	GALK2, IRS2, GBE1, ATF3, PFKFB4, ALDOC, HK2, ENO2, PGK1	1.89E-02
**GOTERM_BP_FAT**	GO:0005996 monosaccharide metabolic process	GALK2, IRS2, GBE1, ATF3, PFKFB4, ALDOC, HK2, ENO2, PGK1	2.66E-02
**GOTERM_BP_FAT**	GO:0001666 response to hypoxia	ADM, ALDOC, VEGFA, BNIP3, CITED2, VLDLR, DDIT4	0.0490
**GOTERM_BP_FAT**	GO:0070482 response to oxygen levels	ADM, ALDOC, VEGFA, BNIP3, CITED2, VLDLR, DDIT4	0.04855

Appendix Table S2. Gene Ontology analysis for Human genes highly modulated by COMBO.

Up-Modulated

Category	GO Term	Genes	p value BH corrected
**GOTERM_BP_FAT**	GO:0006955 immune response	CXCL1, CSF2, IFIH1, CCL2, ENPP2, RSAD2, IFI44L, OAS1, OAS2, HLA-DMA, IFI35, CXCL10, LOC100133678, HLA-DRB4, CFH, CFI, DHX58, IL8, BST2, CFB, PRG4, TRIM22, HLA-DQA1, OASL, IL18BP, GBP2, IFI6, HLA-DRA	1.37E-08
**GOTERM_BP_FAT**	GO:0009615 response to virus	PLSCR1, IFIH1, BST2, ISG15, IRF7, RSAD2, MX1, STAT1, TRIM22, IFI35, STAT2	6.66E-06
**GOTERM_BP_FAT**	GO:0006952 defense response	CXCL1, IFIH1, A2M, CCL2, AIF1, RSAD2, CXCL10, CFH, SERPINA3, CFI, MX1, DHX58, GNG7, NFKBIZ, IL8, GAGE2A, CFB, GAGE2E, GAGE2B, GAGE12C, GAGE12B, EPHA3, IRF7, GAGE6, GAGE8, HLA-DRA	9.10264E-05
**GOTERM_BP_FAT**	GO:0009611 response to wounding	CXCL1, NFKBIZ, A2M, CCL2, IL8, AIF1, ERBB3, CFB, COL3A1, EPHA3, CXCL10, PLSCR1, IRF7, SERPINA3, CFH, VCAN, ID3, CFI	0.0001885
**GOTERM_BP_FAT**	GO:0006954 inflammatory response	CXCL1, NFKBIZ, A2M, CCL2, IL8, CFB, AIF1, EPHA3, CXCL10, IRF7, CFH, SERPINA3, CFI	0.001845
**GOTERM_BP_FAT**	GO:0009612 response to mechanical stimulus	CCL2, MGP, RCAN1, COL1A1, STAT1, MMP13	0.01299

Down-Modulated

Category	GO Term	Genes	p value BH corrected
**GOTERM_BP_FAT**	GO:0070482 response to oxygen levels	CA9, ADM, ANG, HMOX1, ALDOC, CITED2	1.64E-03
**GOTERM_BP_FAT**	GO:0001666 response to hypoxia	CA9, ADM, ANG, HMOX1, ALDOC, CITED2	2.55E-03
**GOTERM_BP_FAT**	GO:0009719 response to endogenous stimulus	IRS2, CA9, ADM, ANG, HMOX1, ALDOC, ENO2	0.01596
**GOTERM_BP_FAT**	GO:0010033 response to organic	IRS2, CA9, ADM, ANG, HMOX1, ALDOC, ENO2, HSPA6	0.03828

	substance

Appendix Table S3. Gene Ontology analysis for Mouse genes highly modulated by PLX4720

Down-Modulated

Category	GO Term	Genes	p value<br>BH corrected
**GOTERM_BP_FAT**	GO:0001525 angiogenesis	RTN4, EMCN, PDGFA, PGF, CSPG4, VASH1, MAP3K7, OVOL2, VEGFA, HBEGF, RBPJ, ANGPT2, POFUT1	2.23E-02
**GOTERM_BP_FAT**	GO:0048514 blood vessel morphogenesis	RTN4, EMCN, PGF, PDGFA, PRRX1, CSPG4, VASH1, CITED2, MAP3K7, OVOL2, VEGFA, HBEGF, FOXC1, RBPJ, ANGPT2, POFUT1	3.72E-02
**GOTERM_BP_FAT**	GO:0001944 vasculature development	RTN4, EMCN, TBX3, PGF, PDGFA, PRRX1, CSPG4, VASH1, CITED2, MAP3K7, OVOL2, VEGFA, HBEGF, FOXC1, RBPJ, ANGPT2, POFUT1	0.03856
**GOTERM_BP_FAT**	GO:0001568 blood vessel development	RTN4, EMCN, TBX3, PGF, PDGFA, PRRX1, CSPG4, VASH1, CITED2, MAP3K7, OVOL2, VEGFA, HBEGF, FOXC1, RBPJ, ANGPT2, POFUT1	0.03879

Appendix Table S4. Gene Ontology analysis for Mouse genes highly modulated by COMBO.

Up-Modulated

Category	GO Term	Genes	p value BH corrected
**GOTERM_BP_FAT**	GO:0006955 immune response	CIITA, GBP6, CD8B1, C3, H2-M2, LY86, IL18, H2-D1, TLR2, SAMHD1, IGH-6, OAS2, CCL5, H2-Q7, PSMB9, OSM, PSEN2, IL1B, XCL1, CFD, LTB, CD14, H28	6.74E-08
**GOTERM_BP_FAT**	GO:0001819 positive regulation of cytokine production	CD83, IL18, KLRE1, TLR2, IL1B, H2-Q7, CD14	5.05E-03
**GOTERM_BP_FAT**	GO:0051240 positive regulation of multicellular organismal process	CD83, SLC1A3, IL18, PSEN2, KLRE1, TLR2, IL1B, H2-Q7, LTB, CD14	5.62E-03
**GOTERM_BP_FAT**	GO:0010033 response to organic substance	CIITA, GATM, TLR2, STAT1, CCL5, ADIPOQ, CD48, FOS, CD83, SLC1A3, APOE, IL1B, SORT1, NFE2L2, CD14, NR1H3	6.72E-03
**GOTERM_BP_FAT**	GO:0009611 response to wounding	CIITA, GATM, NFKBID, C3, LY86, TLR2, NINJ1, CHI3L3, CCL5, SLC1A3, IL1B, CFD, CD14	8.63E-03
**GOTERM_BP_FAT**	GO:0006954 inflammatory response	CIITA, NFKBID, C3, LY86, TLR2, IL1B, CHI3L3, CFD, CCL5, CD14	2.32E-02
**GOTERM_BP_FAT**	GO:0001817 regulation of cytokine production	CD83, IL18, KLRE1, TLR2, IL1B, H2-Q7, LTB, CD14	2.81E-02
**GOTERM_BP_FAT**	GO:0002684 positive regulation of immune system process	CD83, C3, IL18, PSEN2, KLRE1, TLR2, IGH-6, CFD, H2-Q7	4.99E-02

Down-Modulated

Category	GO Term	Genes	p value BH corrected
**GOTERM_BP_FAT**	GO:0022610 biological adhesion	AEBP1, EMCN, BCAR1, TNC, FERMT2, CLDN5, CDH5, VCL, PCDH1, COL6A2, COL6A1, ESAM, COL18A1, ICAM2, COL15A1, HSPG2, PCDH12, ITGA3, NID1, NID2, MCAM, COL5A1, NCAM1, VWF, CDH13, ITGA5, CPXM1, PECAM1, LAMC1, PARVB	7.09E-10
**GOTERM_BP_FAT**	GO:0007155 cell adhesion	AEBP1, EMCN, BCAR1, TNC, FERMT2, CLDN5, CDH5, VCL, PCDH1, COL6A2, COL6A1, ESAM, COL18A1, ICAM2, COL15A1, HSPG2, PCDH12, ITGA3, NID1, NID2, MCAM, COL5A1, NCAM1, VWF, CDH13, ITGA5, CPXM1, PECAM1, LAMC1, PARVB	1.36E-09

**GOTERM_BP_FAT**	GO:0001568 blood vessel development	COL18A1, EMCN, FLT1, EGFL7, PGF, GJA1, CDH5, COL5A1, CITED2, MAP3K7, VEGFC, HMOX1, NOTCH4, ROBO4, NOS3, SOX18, COL1A1, LOX, PLXND1, ANGPT2	4.99E-09
**GOTERM_BP_FAT**	GO:0001944 vasculature development	COL18A1, EMCN, FLT1, EGFL7, PGF, GJA1, CDH5, COL5A1, CITED2, MAP3K7, VEGFC, HMOX1, NOTCH4, ROBO4, NOS3, SOX18, COL1A1, LOX, PLXND1, ANGPT2	5.73E-09
**GOTERM_BP_FAT**	GO:0001525 angiogenesis	COL18A1, EMCN, FLT1, EGFL7, PGF, MAP3K7, VEGFC, HMOX1, NOTCH4, ROBO4, NOS3, SOX18, PLXND1, ANGPT2	3.71E-07
**GOTERM_BP_FAT**	GO:0048514 blood vessel morphogenesis	COL18A1, EMCN, FLT1, EGFL7, PGF, GJA1, CITED2, MAP3K7, VEGFC, HMOX1, NOTCH4, ROBO4, NOS3, SOX18, PLXND1, ANGPT2	5.84E-07
**GOTERM_BP_FAT**	GO:0051270 regulation of cell motion	COL18A1, KISS1, ACTN4, EGFL7, SEMA3F, BCAR1, PECAM1, ROBO4, TIE1, VCL	3.29E-04
**GOTERM_BP_FAT**	GO:0030334 regulation of cell migration	COL18A1, KISS1, EGFL7, BCAR1, PECAM1, ROBO4, TIE1, VCL	7.69E-03

Appendix Table S5. Top 5 Upstream Regulators selectively characterizing COMBO treatment, predicted by Upstream Regulators Analysis in IPA.

Upstream Regulator	Molecule Type	Predicted Activation State	p value of overlap	Target molecules in dataset
**TNF**	cytokine		3,01E-33	ACKR2,AEBP1,APOE,CD14,CDC42EP5,CDH5, CDO1,CFD,CIITA,COL15A1,COL1A1,CYP2E1, EDNRB,EFNA1,EMP1,ESM1,F2RL1,F2RL3,FP R2,FSCN1,GJA1,GPD2,GPR176,HLA- A,HMOX1,HP,HSD17B7,HSPA1A/HSPA1B,HS PA4,HSPG2,ICAM2,IFIT3,IL10RA,IL1B,ITGA5,L AMC1,LOX,LTB,MCAM,NCAM1,NFE2L2,NID1, NINJ1,NME3,NOS3,NOTCH4,NR1H3,OSM,PD E4B,PECAM1,PHLDA1,PLAT,PLK2,PPAP2A,P PP1R3C,PSMB9,RABEP2,RARRES2,RGS1,RO BO1,SEPP1,SPARC,STAT1,TIE1,TLR2,TNC,T WIST2,USP2,VCL,VEGFC,WNT5A
**TGFB1**	growth factor	Inhibited	2,14E-29	ACKR2,ACTA2,ACTG2,ANKRD1,APOE,CCRL2 ,CD14,CDH5,CIITA,COL18A1,COL1A1,COL4A1 ,COL4A2,COL5A1,COL6A1,COL6A2,COL6A3,C PXM1,DLL3,EDNRB,ESAM,ESM1,F2RL1,FAM1 10B,FERMT2,FLT1,FPR2,FSCN1,GATM,GJA1, GPRC5B,HMOX1,HOXA2,HS6ST2,HSPA1A/HS PA1B,HSPG2,ICAM2,IFIT3,IL10RA,IL1B,IMPDH 1,ITGA3,ITGA5,LAMC1,LOX,NCAM1,NOS3,NR 1H3,OSM,PECAM1,PILRA,PLAT,PLK2,PPP1R3 C,PTPRK,RAMP2,SAMHD1,SEMA7A,SMTN,SP ARC,STAT1,TLR2,TNC,TPM1,TWIST2,VCL,VE GFC,WNT5A
**lipopolysaccharide**	chemical drug		2,01E-23	ABCC5,ACTA2,APOE,CCNL1,CCRL2,CD14,CD 276,CD33,CDH5,CFD,CIITA,CLDN5,COL1A1,C OL4A1,COL4A2,COL5A1,COL6A1,CTSL,CYP2 E1,F2RL1,FLT1,FPR2,FSCN1,GJA1,H2- M2,HLA- A,HMOX1,HP,HSPA1A/HSPA1B,ICAM2,IFIT3,I L10RA,IL1B,ITGA5,LYZ,MYL9,NID1,NOS3,NR1 H3,OSM,PDE4B,PECAM1,PHLDA1,PLAT,PLK2

			,PSMB9,RASL11A,RGS1,SATB1,SEPP1,SEPT1,SLCO2A1,SPARC,STAT1,TCAP,TLR2,TNC,TOR3A,VCL,VEGFC,XCL1
**dexamethasone**	chemical drug	9,41E-17	ACTA2,ACTG2,ADRBK2,AEBP1,ANKRD1,APOE,BCAR1,CFD,CLDN5,COL15A1,COL1A1,COL4A1,COL4A2,COL6A1,COL6A2,CPE,CTSL,CYP2E1,EDNRB,EFNA1,F2RL1,GJA1,GPR65,HP,HSD17B7,IFIT3,IL10RA,IL1B,ITGA5,LAMC1,LTB,NCAM1,NR1H3,OSM,PDE4B,PHLDA1,PLAT,PLK2,RGS1,SEPP1,SLCO2A1,SLCO2B1,SPARC,STAT1,TLR2,TNC,TPM1,VCL,VEGFC,WNT5A,XCL1
**IL1B**	cytokine	1,16E-15	ACKR2,ACTA2,APOE,CCRL2,CD14,CIITA,COL1A1,CYP2E1,EFNA1,F2RL1,FLT1,FPR2,GJA1,HLA-A,HMOX1,HP,HSPA1A/HSPA1B,HSPG2,IFIT3,IL10RA,IL1B,ITGA5,LOX,NFE2L2,NOS3,NR1H3,OSM,PDE4B,PHLDA1,PLAT,PSMB9,SEPP1,SPARC,STAT1,TLR2,VEGFC

Appendix Table S6. Primers Used for Quantitative Real Time PCR Expression Analysis

**GM-CSF**	5’-GGGAGCATGTGAATGCCATC-3’	5’-GGCTCCTGGAGGTCAAACAT-3’	Human
**TBP**	5’-GTGACCCAGCATCACTGTTTC-3’	5’-GAGCATCTCCAGCACACTCT-3’	Human
**TNF**	5’-CCCATGTTGTAGCAAACCCTC-3’	5’-TATCTCTCAGCTCCACGCCA-3’	Human
**TGFB1**	5’- CGTGGAGGGGAAATTGAGGG-3’	5’- CCGGTAGTGAACCCGTTGATG-3’	Human
**Arg1**	5’-TTTTAGGGTTACGGCCGGTG-3’	5’-CCTCGAGGCTGTCCTTTTGA-3’	Mouse
**Ccl5**	5’-GACAGCACATGCATCTCCCA-3’	5’-GTGTCCGAGCCATATGGTGA-3’	Mouse
**Cd40**	5’-TTGTTGACAGCGGTCCATCT-3’	5’-TCTCAAGAGCTGTGCAGTGG-3’	Mouse
**Cdh5**	5’-CCTGAGGCAATCAACTGTGC-3’	5’-GGAGGAGCTGATCTTGTCCG-3’	Mouse
**Col4a1**	5’-GGCCCTTCATTAGCAGGTGT-3’	5’-GTGAGGACCAACCGTTAGGG-3’	Mouse
**Col4a2**	5’-CGGCGTAATCTCAAAAGGCG-3’	5’-GGCCTCTGCTTCCTTTCTGT-3’	Mouse
**Cxcl10**	5’-GAGAGACATCCCGAGCCAAC-3’	5’-GGGATCCCTTGAGTCCCAC-3’	Mouse
**Cxcl9**	5’-TGGAGTTCGAGGAACCCTAGT-3’	5’-TTGTAGTGGATCGTGCCTCG-3’	Mouse
**Flt1**	5’-TACCTCACCGTGCAAGGAAC-3’	5’-AAGGAGCCAAAAGAGGGTCG-3’	Mouse
**Hey1**	5’-TGCAGTTAACTCCTCCTTGCC-3’	5’-CGCCGAACTCAAGTTTCCATT-3’	Mouse
**Il10**	5’-GGCGCTGTCATCGATTTCTC-3’	5’-ATGGCCTTGTAGACACCTTGG-3’	Mouse
**Il1β**	5’-TGCCACCTTTTGACAGTGATG-3’	5’-TGATGTGCTGCTGCGAGATT-3’	Mouse
**Nfkb1**	5’-GGTCACCCATGGCACCATAA-3’	5’-GGGCCCTTCACCTTCAGTTT-3’	Mouse
**Pdgfb**	5’-GGAGTCGGCATGAATCGCT-3’	5’-GCCCCATCTTCATCTACGGA-3’	Mouse
**Pecam**	5’-AGCCAACAGCCATTACGGTTA-3’	5’-AGCCTTCCGTTCTCTTGGTG-3’	Mouse
**Ptn**	5’-CCGCCTTGAAGACCAGAACT-3’	5’-TTTGACTCCGCTTGAGGCTT-3’	Mouse
**Stat1**	5’-GATCGCTTGCCCAACTCTTG-3’	5’-ACTGTGACATCCTTGGGCTG-3’	Mouse
**Tbp**	5’-AGTGCCCAGCATCACTATTTCA-3’	5’-GCCCTGAGCATAAGGTGGAA-3’	Mouse
**Tgfb1**	5’-AGCTGCGCTTGCAGAGATTA-3’	5’-AGCCCTGTATTCCGTCTCCT-3’	Mouse
**Tlr2**	5’-AAGGAGGTGCGGACTGTTTC-3’	5’-GAGATTTGACGCTTTGTCTGAGG-3’	Mouse

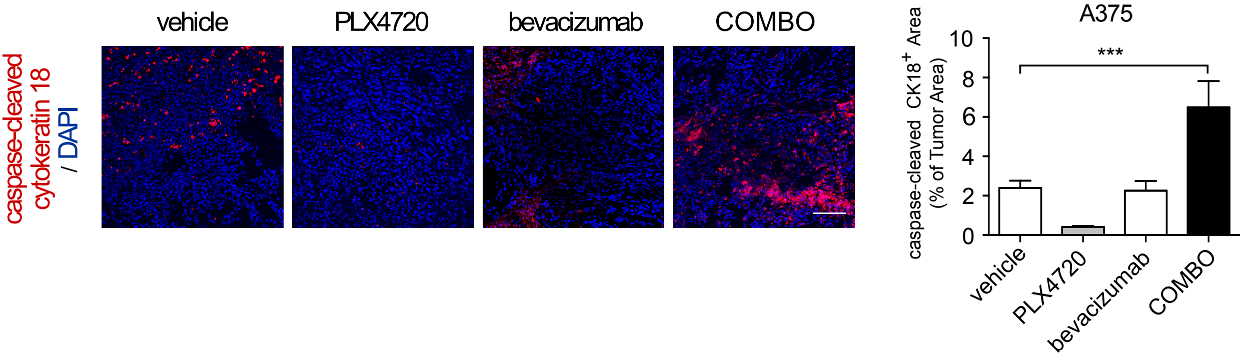

vehicle
PLX4720
bevacizumab
COMBO
caspase-cleaved cytokeratin 18 / DAPI
A375
caspase-cleaved CK18+ Area (% of Tumor Area)
10
8
6
4
2
0
***
vehicle
PLX4720
bevacizumab
COMBO

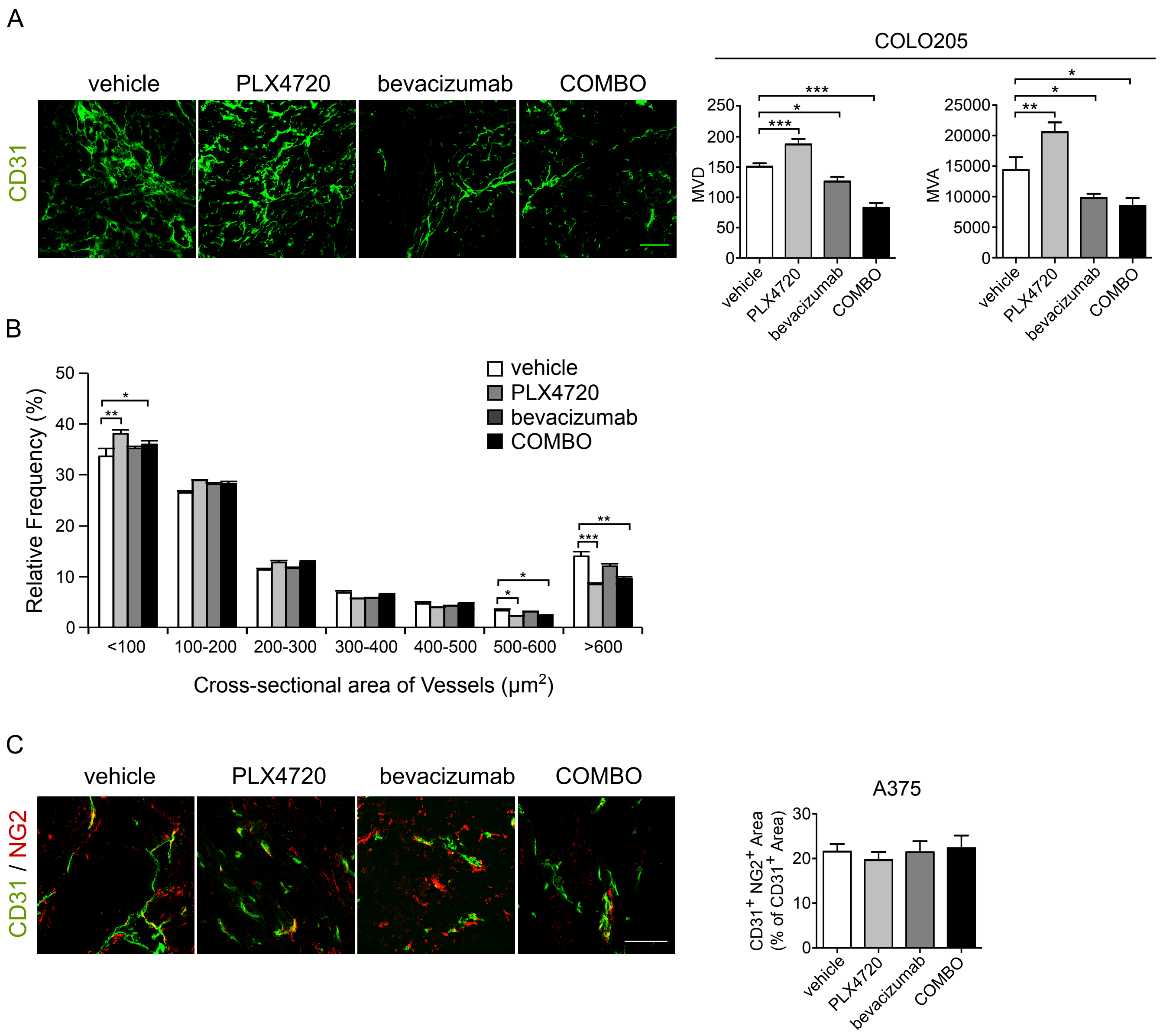
A
vehicle
PLX4720
bevacizumab
COMBO
CD31
COLO205
MVD
MVA
***
*
***
**
0
50
100
150
200
250
5000
10000
15000
20000
25000
B
Relative Frequency (%)
Cross-sectional area of Vessels (μm²)
<100
100-200
200-300
300-400
400-500
500-600
>600
C
CD31 / NG2
A375
CD31+ NG2+ Area
(% of CD31+ Area)

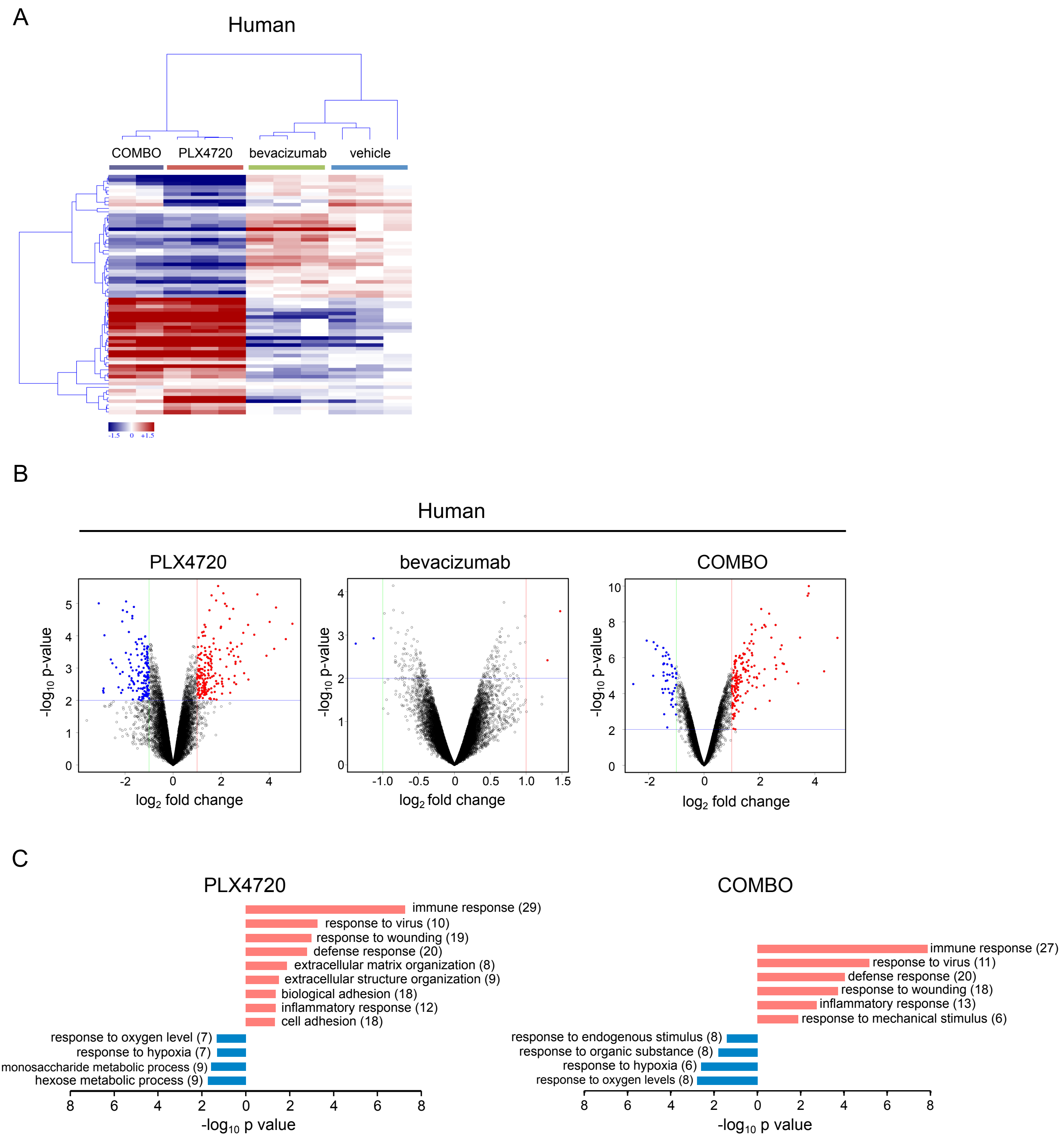
A
Human
COMBO
PLX4720
bevacizumab
vehicle
-1.5 0 +1.5
B
Human
PLX4720
bevacizumab
COMBO
-log10 p-value
log2 fold change
C
PLX4720
immune response (29)
response to virus (10)
response to wounding (19)
defense response (20)
extracellular matrix organization (8)
extracellular structure organization (9)
biological adhesion (18)
inflammatory response (12)
cell adhesion (18)
response to oxygen level (7)
response to hypoxia (7)
monosaccharide metabolic process (9)
hexose metabolic process (9)
-log10 p value
COMBO
immune response (27)
response to virus (11)
defense response (20)
response to wounding (18)
inflammatory response (13)
response to mechanical stimulus (6)
response to endogenous stimulus (8)
response to organic substance (8)
response to hypoxia (6)
response to oxygen levels (8)
-log10 p value

A

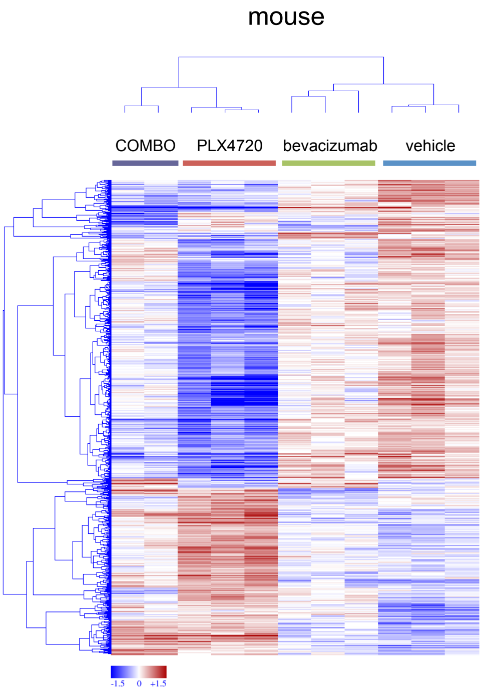

B

Gene	logFC	p value
Ptn	-1.28	0.00
Flt1	-0.86	0.00
Pecam	-0.74	0.00
Col4a1	-0.69	0.00
Cdh5	-0.79	0.00
Sema3f	-0.89	0.00
Flt4	-0.80	0.01
Col18a1	-0.58	0.01
Cola4a2	-0.66	0.01
Thbs1	-0.79	0.02
Hey1	-0.51	0.03
Pdgfb	-0.48	0.03
Hspg2	-0.49	0.03
Cxcl12	0.65	0.04
Fst	-0.62	0.04
F2	-0.41	0.04

C

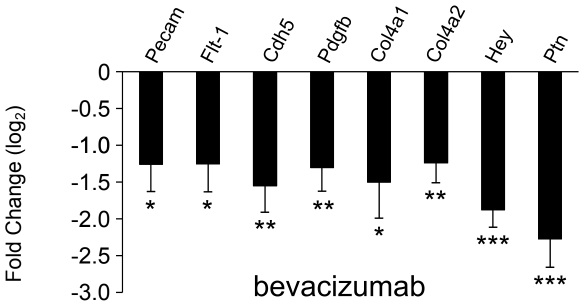

D

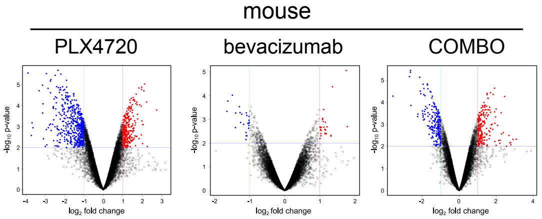

E

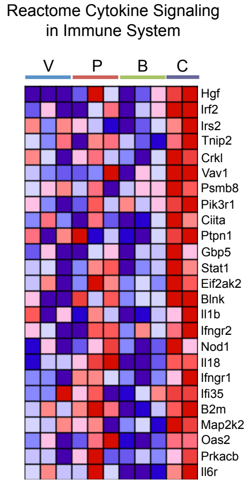

F

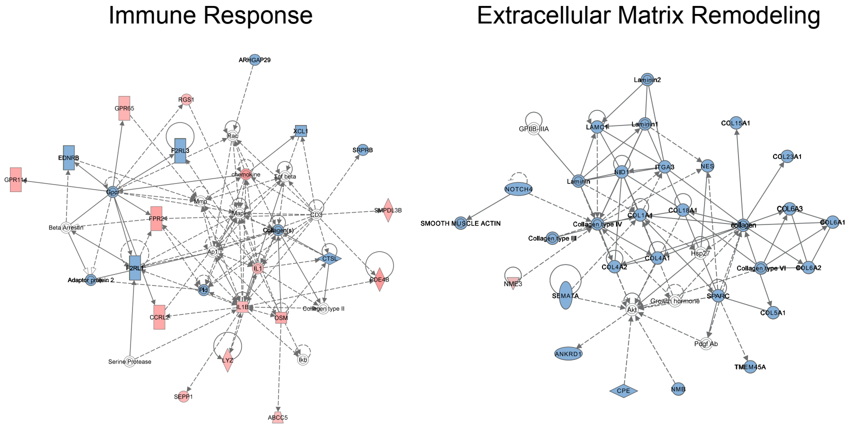

A

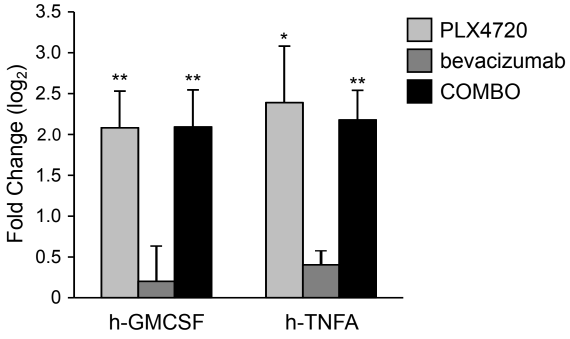

Fold Change (log2)
3.5
3.0
2.5
2.0
1.5
1.0
0.5
0
**
**
*
**
h-GMCSF
h-TNFA
PLX4720
bevacizumab
COMBO

B

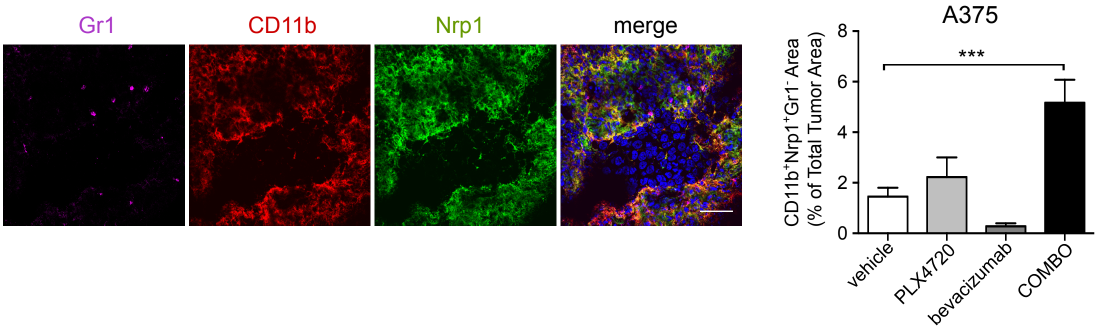

Gr1
CD11b
Nrp1
merge
A375
CD11b+Nrp1+Gr1- Area
(% of Total Tumor Area)
8
6
4
2
0
***
vehicle
PLX4720
bevacizumab
COMBO

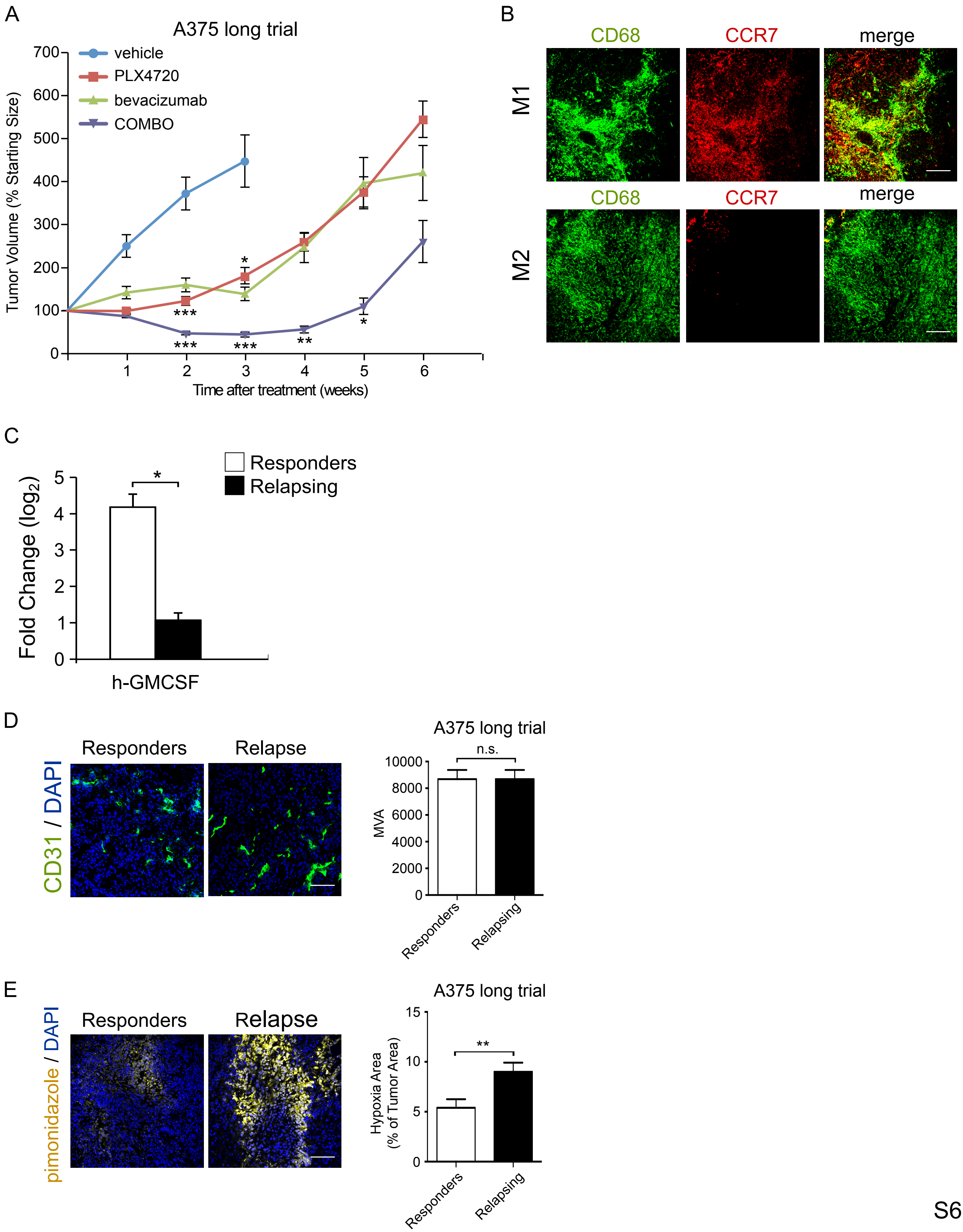
A
A375 long trial
vehicle
PLX4720
bevacizumab
COMBO
Tumor Volume (% Starting Size)
Time after treatment (weeks)
B
CD68
CCR7
merge
M1
M2
C
Responders
Relapsing
Fold Change (log2)
h-GMCSF
D
Responders
Relapse
CD31 / DAPI
A375 long trial
n.s.
MVA
Responders
Relapsing
E
Responders
Relapse
pimonidazole / DAPI
A375 long trial
Hypoxia Area (% of Tumor Area)
Responders
Relapsing
