# Supplementary figures and images for: VEGF blockade enhances the antitumor effect of BRAFV 600E inhibition
Source: EMBO Mol Med. 2016 Dec 14;9(2):219–37. doi: 10.15252/emmm.201505774 (PMC5286370; doi:10.15252/emmm.201505774)

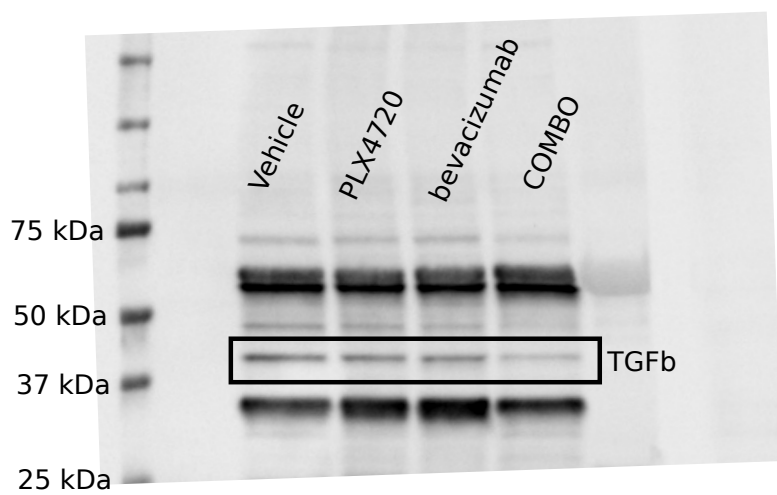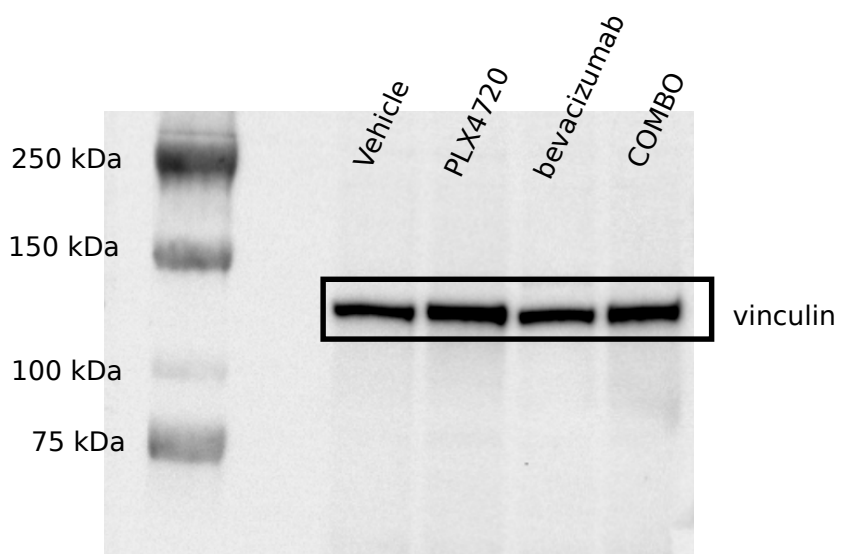

Supplement: Supplementary file 3 — Source Data for Figure 6 [file EMMM-9-219-s002.pdf]
